# Electrical stimulation of the vestibular nerve: evaluating effects and potential starting points for optimization in vestibular implants

**DOI:** 10.1097/MOO.0000000000001001

**Published:** 2024-08-29

**Authors:** Marieke ten Hoor, Raymond van de Berg, Angélica Pérez Fornos, Joost Johannes Antonius Stultiens

**Affiliations:** a Department of Otorhinolaryngology & Head and Neck Surgery, School for Mental Health and Neuroscience, Faculty of Health Medicine and Life Sciences, Maastricht University Medical Center, Maastricht, The Netherlands; b Service of Otorhinolaryngology and Head and Neck Surgery, Department of Clinical Neurosciences, Geneva University Hospitals, Geneva, Switzerland

**Keywords:** bilateral vestibulopathy, neuroprosthetics, semicircular canals, vestibular implant, vestibulo-ocular reflex

## Abstract

**Purpose of review:**

Oscillopsia and unsteadiness are common and highly debilitating symptoms in individuals with bilateral vestibulopathy. A lack of adequate treatment options encouraged the investigation of vestibular implants, which aim to restore vestibular function with motion-modulated electrical stimulation. This review aims to outline the ocular and postural responses that can be evoked with electrical prosthetic stimulation of the semicircular canals and discuss potential approaches to further optimize evoked responses. Particular focus is given to the stimulation paradigm.

**Recent findings:**

Feasibility studies in animals paved the way for vestibular implantation in human patients with bilateral vestibulopathy. Recent human trials demonstrated prosthetic electrical stimulation to partially restore vestibular reflexes, enhance dynamic visual acuity, and generate controlled postural responses. To further optimize prosthetic performance, studies predominantly targeted eye responses elicited by the vestibulo-ocular reflex, aiming to minimize misalignments and asymmetries while maximizing the response. Changes of stimulation parameters are shown to hold promise to increase prosthetic efficacy, together with surgical refinements and neuroplastic effects.

**Summary:**

Optimization of the stimulation paradigm, in combination with a more precise electrode placement, holds great potential to enhance the clinical benefit of vestibular implants.

## INTRODUCTION

The vestibular organ functions as a biomechanical sensor responsible for the detection of head motion. It comprises three semicircular canals, which are sensitive to angular motion, and two otolith organs, which are mainly sensitive to linear motion and tilt [1]. Through a synergy of reflexes, vestibular input is integral to gaze stabilization, postural control and spatial orientation. As these functionalities are essential for daily life, their impairment due to vestibulopathy can be severely debilitating.

Approximately 1.8 million adults worldwide suffer from severe bilateral vestibulopathy (BVP), with about half having no identified cause [2–4]. Common identified causes include ototoxicity, Menière's disease, genetic disorders and meningitis [5,6]. Affected individuals often report oscillopsia (unstable vision during head movements) and unsteadiness [4]. These symptoms can greatly interfere with otherwise routine activities, like walking and driving, and typically worsen in the dark and on uneven terrain [7,8]. BVP significantly impairs physical and social functioning, reducing quality of life [9,10]. Its prognosis is generally poor, as most patients’ vestibular function does not improve, regardless of cause [3]. Although vestibular rehabilitation therapy is the treatment of choice, providing an exercise-based program designed to promote vestibular adaptation and substitution, outcomes vary between patients and benefits are typically limited to slow and predictable movements [6,11,12].

To address this lack of adequate treatment, and inspired by the success of cochlear implants, the feasibility of restoring vestibular function with electrical stimulation is currently being investigated. Two different methodologies are pursued, targeting the semicircular canals [13,14] or otolith organs [15,16] as the stimulation site. As the semicircular canals are responsible for eliciting the vestibulo-ocular reflex (VOR), the former approach holds potential to treat oscillopsia. The three semicircular canals, oriented nearly orthogonally, are most sensitive to head rotations parallel to their respective planes. This arrangement yields a three-dimensional representation of head motion, which enables the VOR to generate compensatory eye movements to stabilize gaze. It also allows for site-specific electrical stimulation to encode distinct directions of head movement, which renders the semicircular canals well suited for prosthetic implantation. Therefore, this review will primarily focus on the restoration of semicircular canal function.

It was first demonstrated in animal models that electrical stimulation of ampullary nerves can evoke eye and head movements, parallel to the plane of the innervated semicircular canal [17–19]. Building on this concept, a single-channel vestibular implant prototype was proposed [20,21] and developed further by incorporating motion sensors and multichannel electrode leads [22]. After demonstrating the ability to partially restore the VOR in animals with inflicted vestibular damage, the first experiments in humans were conducted. Electrical stimulation of ampullary nerves could evoke eye movements parallel to the plane of the stimulated posterior [23], anterior, and lateral semicircular canal [24]. This encouraged the development and implantation of single-channel [25], followed by multichannel prostheses in patients with BVP [14,26–29]. Efficacy studies demonstrated partial restoration of vestibular reflexes [26,29–32], enhancement of dynamic visual acuity (DVA) [33,34], and generation of controlled postural responses [27,30]. This review aims to outline the ocular and postural responses that can be evoked with electrical stimulation of ampullary nerve endings and how these can potentially be improved, with particular focus on the stimulation paradigm. 

**Box 1:**
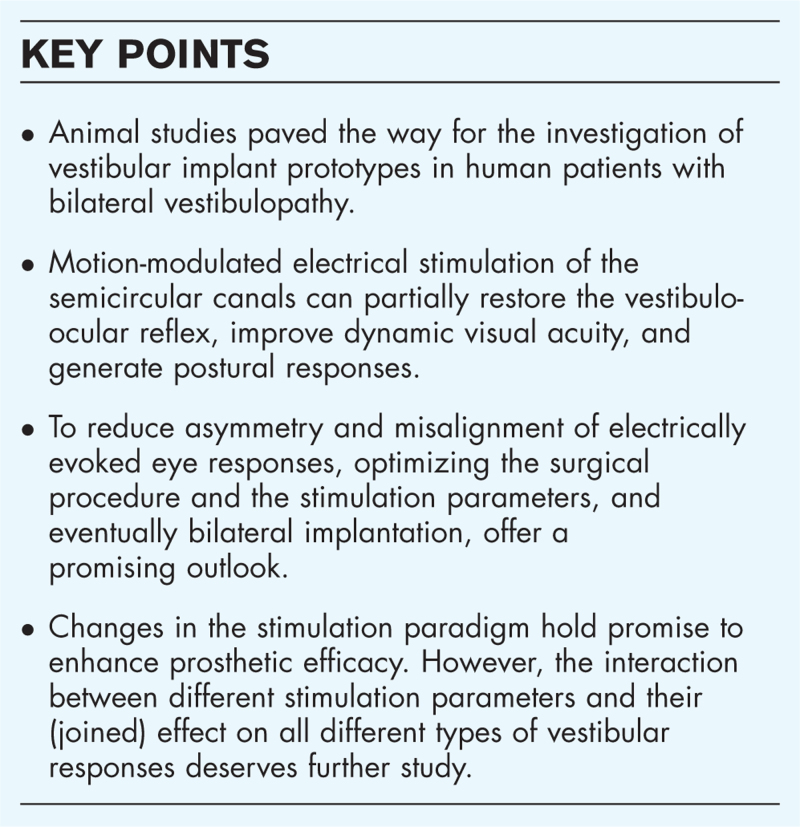
no caption available

## THE CONCEPT OF A VESTIBULAR IMPLANT

**FIGURE 1:**
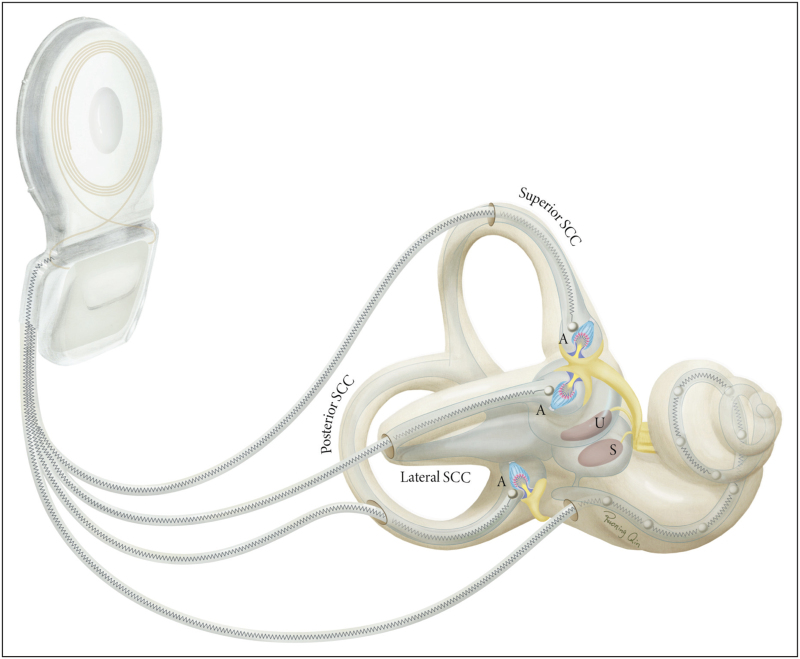
Vestibular implant integrated with a cochlear implant. Three electrode leads target ampullary nerve endings in the posterior, anterior and lateral semicircular canals (SCCs). The fourth electrode lead accommodates electrical stimulation sites in the cochlea. Electrical stimulation is controlled by a pulse generator that communicates with the externally worn motion processor (not shown) through inductively coupled coils (only the internal/receiving coil is shown). A = ampulla, U = utricle, S = saccule. Reproduced with permission from Stultiens *et al.*[82]. Illustration made by Ruoning Qin.

The purpose of vestibular implants is to artificially restore vestibular function in patients with BVP, by providing the central nervous system with head motion cues using electrical stimulation of vestibular nerve branches. This is analogous to the cochlear implant, which aims to restore hearing function by electrically stimulating the auditory nerve. Hence, the vestibular implant design is similar to, and may even be integrated with, a cochlear implant design (Fig. 1). The vestibular implant is equipped with a sensing unit to capture head motion (e.g. gyroscope and accelerometer) and a processor to convert motion data (i.e. orientation and velocity) into stimulation patterns. Based on these patterns, a pulse generator modulates a train of biphasic electrical pulses and activates electrodes that are surgically placed in the vicinity of ampullary nerve endings [26,29].

As the vestibular system's neural infrastructure is specifically tailored to the processing of natural inputs, mimicking these in the electrical stimulation pattern is deemed the most credible approach to restore vestibular function. In the healthy system, ampullary nerves up-modulate or down-modulate their firing rate relative to a baseline to encode bidirectional head rotations. To restore this bidirectional sensitivity with electrical stimulation, a baseline stimulation (constant pulse rate and amplitude) can be implemented. Following an adaptation period, the stimulation intensity can be up-modulated or down-modulated relative to this baseline stimulation [25,35]. The maximum intensity available for modulation is typically limited by the tolerance of the user (i.e. upper comfortable level) or by the activation of nontargeted nerves. This level may vary significantly between electrodes and between users, consistent with findings in other neuroprosthetics [36]. Stimulus intensities available for modulation can be mapped to angular head velocities in three-dimensional space, with each dimension assigned to a different semicircular canal. When motion-modulated stimulation is turned on, the vestibular implant activates its electrodes based on real-time head movements and preconfigured mappings to generate vestibular responses.

## EVALUATING VESTIBULAR IMPLANT PERFORMANCE

Most studies used the electrically evoked VOR response to evaluate the performance of semicircular canal implants, providing an objective measure that is relatively simple to assess and monitor through repeated measures. VOR responses can be quantified by gain and phase. While gain refers to the amplitude ratio of head and eye movements, phase denotes their relative timing. VOR deficiencies typically result in a decreased amplitude and delayed timing of compensatory eye responses, reflected by impaired gain and phase. As this deficiency presumably induces oscillopsia, which is a primary complaint among patients with BVP, VOR restoration is a key objective in vestibular rehabilitation [4].

The head impulse test is a widely used clinical tool to evaluate VOR function, with VOR gain as the main outcome measure [37,38]. Participants are instructed to fixate their gaze on a stationary target while the examiner generates brief and high-velocity head impulses. The high speed prevents nonvestibular oculomotor control mechanisms from generating compensatory eye movements [39]. By aligning the head impulses with the approximate plane of two opposite canals, their function can be studied selectively. Head impulses in one direction will lead to strong excitation of the ipsilateral canal and weaker inhibition of the contralateral canal. In the video head impulse test (vHIT), eye movements are simultaneously recorded.

In vestibular implant recipients, vHIT experiments showed that motion-modulated electrical stimulation could partially restore the high-frequency VOR [31]. Increased stimulation intensity improved median VOR gain for excitatory head impulses in all participants and for inhibitory head impulses in some participants. Excitatory stimulation generally evoked larger responses than inhibitory stimulation, consistent with asymmetries innate to the vestibular system [40–42]. However, not all electrodes could evoke eye responses aligned with the respective stimulated canal. Complementary to vHIT, horizontal rotary chair experiments were performed using sinusoidal rotations up to 2 Hz [26,29]. Motion-modulated stimulation significantly improved VOR gain in all participants for 0.1–0.5 Hz rotations and in all but one participant for 1–2 Hz rotations, and achieved significantly higher VOR gain as compared with baseline stimulation [29]. The electrically evoked VOR response increased with rotation frequency, reaching maximum VOR gain for 1–2 Hz rotations [26], consistent with the frequency dependency of the natural vestibular system [32,43,44]. These findings suggest that vestibular implants are effective across a functionally relevant frequency range, particularly because head movements at 1–2 Hz are common in essential everyday activities like walking [45,46]. Additionally, the longer response times of the visual and somatosensory systems highlight the crucial role of vestibular inputs in stabilizing gaze during these high-frequency head movements [47].

Other studies investigated DVA to functionally assess vestibular implant performance. DVA is assumed to indirectly reflect VOR function. Deficient VOR gain leads to retinal image slip [37,48–50], which may compromise DVA when its velocity exceeds 2–4° per second [51,52]. Yet, other vestibular reflexes may also contribute. One study tested the ability to read Sloan letters while walking on a treadmill and demonstrated that vestibular implant recipients performed significantly better with coherent motion-modulation stimulation compared with no or reversed motion-modulated stimulation [33]. A case study investigated high-frequency DVA using a functional HIT, where the seated vestibular implant user was instructed to identify briefly displayed optotypes during passive high-velocity head impulses [34]. In the horizontal plane that was tested, better results were observed compared with no or reversed motion-modulated stimulation. Interestingly, this was the case with both baseline and with coherent motion-modulated stimulation. However, the latter yielded the greatest improvement [34].

Motion-modulated electrical stimulation also holds promise to enhance postural control, as indicated by various gait and balance measures [53]. Improvements relative to preoperative assessments and comparisons with placebo stimulation suggested that observed effects were not simply caused by placebo or spontaneous recovery. Furthermore, measured cervical vestibular-evoked myogenic potentials implied activation of vestibular-collic pathways [30,54] and controlled whole-body postural responses suggested activity of vestibulo-spinal pathways [55].

To provide a viable treatment option, vestibular implants must maintain efficacy over extended periods of use. A study in patients with Menière's disease reported that VOR responses decayed over subsequent sessions of electrical stimulation [56,57]. Similarly, long-term depression was observed in central vestibular neurons of nonhuman primates, suppressing vestibulo-ocular and vestibulo-spinal responses [58,59]. However, these decays may be partly explained by the lack of vestibular stimulation and head movement between test sessions [58]. Another study in humans with BVP showed that, although VOR responses were suppressed within an hour after vestibular implant activation, VOR responses could still be electrically evoked after extended periods of continuous implant use [29]. In this study population, outcome measures related to gait, posture and quality of life were generally in the direction of improvement after 0.5 and 1 year of continuous motion-modulated electrical stimulation, as compared with preimplantation [53]. Furthermore, improvements in vestibular symptoms, self-perceived disability and quality-of-life scores reported at 0.5 years postimplantation persisted up to 6 years and were greater than those reported by nonimplanted patients with BVP who received standard-of-care treatment (i.e. exercise-based vestibular rehabilitation) [60^▪^].

## TOWARDS A MORE SYMMETRICAL AND ALIGNED EYE RESPONSE

Two important areas for improvement in the electrically evoked VOR response include symmetry and alignment. Symmetry denotes the amplitude ratio of excitatory and inhibitory eye responses, while alignment indicates the directional difference between the achieved and targeted (i.e. parallel to the plane of the stimulated canal) eye response. A common strategy to pursue symmetric responses involves up-modulating and down-modulating the electrical stimulation relative to a baseline, providing bidirectional motion cues to the central nervous system. However, the effect of this approach is limited by asymmetries of the vestibular system, as it is less sensitive to inhibitory stimuli [40,41,61]. Increasing the level of baseline stimulation could somewhat increase inhibitory eye velocity, but it markedly decreased excitatory eye velocity [20,62]. Bilateral vestibular implantation likely holds most promise for achieving symmetric responses. Until this approach gains clinical acceptance, unilateral restoration of vestibular function appears to be a viable option for treating BVP. Supporting this, studies have demonstrated that unilateral vestibular implantation can improve the quality of life for individuals with BVP [53,60^▪^], and unilateral vestibular deficits are generally better compensated for than bilateral deficits [63,64].

Misalignments of the eye response are likely caused by current spread to nontargeted ampullary nerves, especially considering that the ampullary nerves innervating the lateral and anterior semicircular canal are anatomically very close [65,66]. To increase the selectivity of electrical stimulation, electrical current could potentially be ‘steered’ away from nontargeted and towards targeted ampullary nerves [67]. Alternatively, the stimulus waveform could be optimized [66,68]. In animal models, precompensation techniques were also demonstrated to enhance alignment [69]. However, first starting points for improvement likely concern electrode design and placement, as these are key to facilitate an effective yet selective transfer of electricity at the electrode–neuron interface [70,71].

## OPTIMIZATION OF THE STIMULATION PARADIGM

Studies have been exploring the parameter space of the electrical stimulation paradigm to optimize vestibular implant outcomes (Table 1). This approach is considered worthwhile, especially as changes in the encoding strategy of cochlear implants significantly improved speech recognition outcomes [72].

**Table 1:** Changes in the electrically evoked vestibulo-ocular reflex response after manipulation of the electrical stimulation paradigm, duration of continuous use, and electrode position

Topics	Manipulation(s)			Main outcomes	Future considerations
Electrical stimulation	Pulse duration		↓	Less misalignment Larger electrical output range	Investigate combined modulation in humans Further optimize the VOR response Also optimize for postural responses and perceptual outcomes Also consider central contributions of other sensory modalities
	Modulation type	Rate	↑	Lower max. VOR gain Less misalignment at high stimulation intensities	
		Amplitude	↑	Higher max. VOR gain More misalignment at high stimulation intensities	
	Baseline level		↓	Higher max. excitatory VOR gain Less symmetry	
	Sensitivity		↑	Higher VOR gain per velocity Smaller velocity input range	
Neural adaptation	Duration of continuous use		↑	Higher max. VOR gain Less misalignment	Conduct more research on the effect of extended periods of continuous use in humans
Surgery	Electrode placement near target		↑	Higher max. VOR gain Less misalignment	Identify the optimal target for electrode placement Pursue hearing preservation

VOR, vestibulo-ocular reflex.

Most studies suggest smaller pulse widths to be superior to broader pulse widths in terms of efficacy and energy consumption. Smaller pulse widths require less charge to reach activation thresholds and enable a more spatially selective activation of targeted ampullary nerves [66,73,74], maximizing the electrical dynamic range available for stimulation [75]. Theoretically, smaller pulse widths allow for higher pulse rates, which can evoke greater eye responses than lower pulse rates [65]. However, smaller pulse widths generally require higher current levels to achieve the targeted response. As the maximum current amplitude is limited by the implant's electrical capacity and by electrode impedances, the optimal pulse width should be carefully considered [75,76].

**FIGURE 2:**
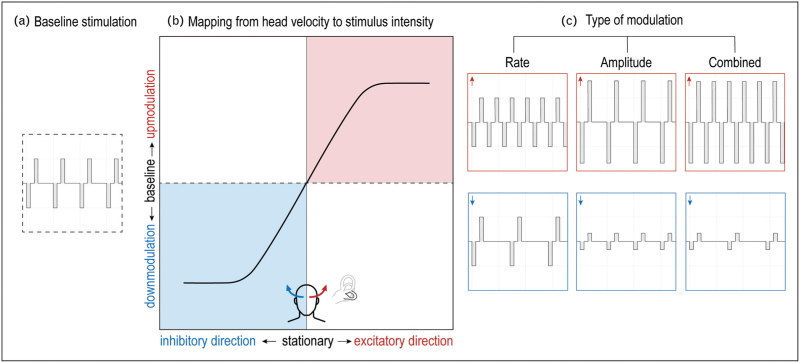
Strategy used by a unilateral vestibular prosthesis to encode bidirectional head movements into electrical stimulation patterns. **A.** Train of biphasic electrical pulses that is provided as baseline stimulation, having a constant pulse amplitude and pulse rate. **B.** Example of a mapping based on which the vestibular prosthesis modulates its electrical stimulation. When the head is stationary (black dashed line), baseline stimulation is provided. When the head turns in the excitatory direction (red), then stimulation intensity is up-modulated. When the head turns in the inhibitory direction (blue), then stimulation intensity is down-modulated. For example, when stimulating the left horizontal semicircular canal (see black outlined canal in grey labyrinth), the excitatory direction corresponds to horizontal turns to the left and the inhibitory direction corresponds to horizontal turns to the right. **C.** The different types of modulation that can be used. Pulse rate and/or pulse amplitude are increased for excitatory movements (red) and decreased for inhibitory movements (blue).

Changing the type of modulation also holds potential to improve stimulation efficacy. Baseline stimulation can be up-modulated and down-modulated in pulse rate, pulse amplitude or a combination thereof (Fig. 2). Animal studies predominantly employed rate modulation, as this strategy best mimics the natural coding of ampullary nerves [13,21,41,69,77]. With increasing stimulation intensity, the alignment of evoked eye responses remained relatively constant with rate modulation but tended to deteriorate with amplitude modulation [62], which is consistent with human studies [78]. In humans, amplitude modulation achieved larger VOR gain than rate modulation, even when employing equivalently charged stimuli [25,78]. Combined modulation yielded maximum VOR gain in animals, but this strategy has not yet been investigated in humans [62].

To further enhance stimulation efficacy, the mapping from head movement to stimulus intensity can be addressed. Mappings that accounted for natural response dynamics of vestibular afferents achieved higher VOR gain and more accurate VOR phase than linear mappings [40,79]. Potentially, VOR gain can be increased further by incorporating a steeper slope in the mapping's linear region, locally increasing the sensitivity of the vestibular implant [31]. However, this may limit the range of head velocities that can be encoded before reaching saturation.

The effects of changing stimulation parameters can be studied by evaluating direct vestibular responses, such as eye responses. In the experiments used, visual and somatosensory inputs can be minimized, for instance, by using stationary and dark conditions. This approach primarily evaluates the specific vestibular contribution. However, these are artificial conditions that likely underestimate the multimodal nature of vestibular responses [80]. Another challenge in the optimization of the stimulation paradigm may be posed by inconsistent responses between different vestibular outcomes (e.g. perception, posture, eye movement) evoked by the same electrical stimulus [27]. Therefore, when tuning the stimulation paradigm of vestibular implants, all different vestibular outcomes should be considered, and responses in more natural circumstances should be evaluated.

## FUTURE DIRECTIONS

The aforementioned findings support the view that varying stimulation parameters may enhance vestibular implant efficacy. It is, however, important to consider that neural adaptivity in central vestibular pathways may already partially compensate for ‘suboptimal’ stimuli, as indicated by changes in eye response amplitude and direction over extended periods of continuous vestibular stimulation [29,77,81]. Further insight into the mechanism of neural adaptivity in humans could potentially provide more direction to the optimization of the stimulation paradigm, by prioritizing the response outcomes that are least compensated for by neural adaptivity.

Recently, additional challenges in developing a clinically effective vestibular implant were described, also highlighting the surgical procedure as an important area for improvement [82]. Minimal electrode repositioning may markedly impact eye response amplitudes and facial nerve activation, emphasizing the need for a precise electrode placement in vestibular implant surgery [24,71]. Ongoing surgical advances provide a promising outlook for a more precise electrode placement. Yet, further research is needed to confirm the optimal target for electrode placement [70,82]. Surgical refinements should also focus on hearing preservation. Currently, vestibular implantation carries great risk of reducing auditory function in the implanted ear [53,56]. This motivated most research groups to combine vestibular implant prototypes with cochlear implants and to only include patients with BVP who are eligible for cochlear implantation [14,15,83,84]. Justifying vestibular implantation in patients with BVP who have residual hearing would significantly expand the target population, likely encompassing a wider range of causes of BVP [85]. Furthermore, larger and more diverse study populations would enable future research to investigate how different causes and long-standing deficits affect the outcomes of vestibular implants. Gained insights could then inform clinical inclusion criteria and aid in managing patient expectations.

## CONCLUSION

In conclusion, vestibular implants hold promise to partially restore semicircular canal function in patients with BVP and to improve their quality of life. Optimization of the electrical stimulation parameters carries great potential to advance the implant's clinical benefit. Further surgical advances are crucial to establish an optimal electrode–neuron interface to deliver electrical stimulation and to expand the potential benefit to a broader patient population.

## Acknowledgements

*None.*

### Financial support and sponsorship

*The authors are representatives of the Geneva-Maastricht research group, which has received support for previous research on vestibular implants, either financially or in-kind, from following agencies: the Dutch government (ZonMw), Med-El (Innsbruck, Austria), Global Education Grant Skolkovo, foundation ‘Stichting Het Heinsius Houbolt Fonds’, and foundation ‘Stichting De Weijerhorst’.*

### Conflicts of interest

*There are no conflicts of interest.*

## REFERENCES AND RECOMMENDED READING

Papers of particular interest, published within the annual period of review, have been highlighted as:

▪ of special interest
▪▪ of outstanding interest
